# From Research to Impact: Factors Shaping Translational Mindset

**DOI:** 10.21203/rs.3.rs-6768753/v1

**Published:** 2025-06-05

**Authors:** Jose Elizondo-González, Trina Emler, Angela Murray

**Affiliations:** University of Kansas; University of Kansas; University of Kansas

**Keywords:** higher education, research translation, SEM, translational mindset, validity

## Abstract

This study investigates the psychological and contextual factors shaping researchers’ translational mindset, conceptualized as a reframing of Academic Entrepreneurial Intention (AEI) and grounded in the Theory of Planned Behavior (TPB). Using the Translational Mindset Scale (TMS), we surveyed 257 researchers and graduate students in engineering, medical, and biological sciences across R1 and R2 institutions in the United States. Structural Equation Modeling (SEM) tested a model in which Translation Intention was predicted by Personal Attraction, Self-Efficacy, and Perceived Social Norms. Results showed that Personal Attraction and Self-Efficacy were strong positive predictors of intention, while Perceived Social Norms had a negative direct effect but a significant positive indirect effect through Self-Efficacy. The model explained 91.5% of the variance in Translation Intention. Contextual variables, such as institutional role and university type, had small but significant effects on motivational constructs. These findings highlight the complex role of institutional expectations and personal motivation in shaping translational engagement, offering guidance for initiatives aimed at strengthening the academic-to-industry research pipeline.

## 1. Introduction

Efforts to promote innovation through academic research have increasingly emphasized the importance of translating findings into applied contexts, including collaboration with industry, policy engagement, and technology transfer. However, researchers’ participation in these activities remains inconsistent, prompting a growing body of literature focused on the psychological and contextual factors that influence such behavior. Much of this work has drawn on the Theory of Planned Behavior (TPB; Ajzen, 1991), which posits that intention is shaped by personal attitude toward the behavior, perceived behavioral control (often operationalized as self-efficacy), and social norms.

Within academic settings, academic entrepreneurial intention (AEI) has surfaced as the major construct operationalization of TPB (Feola et al., 2017; Samo & Huda, 2019). AEI models have provided a useful foundation for understanding why some academics pursue commercialization or venture creation; however, their framing is often grounded in business- and profit-oriented language that often does not resonate with academic researchers. In contrast, many academics view their work as contributing to open knowledge production for societal good rather than as a vehicle for financial gain and, therefore, may perceive entrepreneurial activity as incompatible with scholarly norms (Renault, 2006). While the latter framing aligns with concepts of *social* entrepreneurship, this framing is rarely evidenced in AEI efforts. As such, this semantic tension may discourage engagement from researchers who are otherwise motivated to apply their work beyond academic circles.

Reframing entrepreneurship to language more aligned with academic researchers is one way to address concerns. Translation is a term often used in fields such as biomedical engineering. Translation of research refers to the movement from knowledge-based research or *awareness* into clinical practices or *adoption* of the research (Davis & Taylor-Vaisey, 1997). Despite the wider acceptance of the term *translation*, it, too, has faced barriers in accomplishing its intended goals. Loannidis (2006) states bluntly that “successful translation of research promises is uncommon” (p.1), tracking how published research diminishes to relatively few clinical applications. Green & Seifert (2005) note how little is understood of what is needed to translate from research to adoption of practices or applications.

In response to these compounding issues, Elizondo et al. (under review) proposed the construct of *translational mindset*, grounded in the TPB framework and informed by Liñán and Chen’s (2009) structural model. While preserving the core motivational components of AEI—attitude, control, and norm—translational mindset reframes the construct in language more aligned with academic values and broader forms of application beyond commercialization. It not only builds on AEI but expands its scope to better capture the intentions of those working in environments where open knowledge, social relevance, and non-market outcomes are prioritized. This conceptual shift is reflected in the wording of the Translational Mindset Scale items (TMS; Elizondo et al., under review). For instance, rather than asking whether someone intends to “start a business,” the scale includes items such as *“My professional goal is to translate my scientific research to industry”* (Q5R1) and *“I feel confident in developing a translation project”* (Q4R5), emphasizing intentions and self-efficacy grounded in the academic-to-application pipeline. Similarly, whereas AEI models often focus on social norms shaped by investors or governmental agencies—triple helix models (Leydesdorff & Etzkowitz, 2003)— the TMS measures normative influences more relevant to academic contexts, such as *“Would your unit leadership approve of your decision to translate your scientific research?”* (Q3R4). By anchoring the construct in research translation rather than commercialization, translational mindset offers a more discipline-sensitive framework for understanding how academics are motivated to move their work beyond laboratories into practice, policy, or industry.

Building on this reconceptualization, the present study uses the Translational Mindset Scale to examine the relationships among its core constructs—personal attraction, self-efficacy, and perceived social norms—and their influence on translation intention. Using structural equation modeling (SEM), the study tests a TPB-informed structural model adapted to research translation contexts and explores how contextual variables such as researchers’ institutional role and disciplinary background relate to key motivational pathways. In doing so, this study extends AEI frameworks into a broader, discipline-sensitive model and contributes new insight into the psychological and contextual factors that shape how academic researchers engage with translation.

### 1.1. Entrepreneurial Intention (EI)

In terms of entrepreneurial intention studies, Liñán and Chen (2009) tested several hypotheses using structural equation modeling (SEM) to identify the factors influencing entrepreneurial intention, drawing on the Theory of Planned Behavior (TPB; Ajzen, 1991). In their model, entrepreneurial intention was predicted by three latent constructs: personal attitude toward entrepreneurship, perceived behavioral control (self-efficacy), and subjective norm (perceived social pressure to engage in entrepreneurial behavior). The model also accounted for the potential influence of demographic and human capital variables, including age, nationality, work experience, gender, and the presence of role models. Using a combined sample of Spanish and Taiwanese university students, the authors found that both personal attitude (*β* = 0.663) and perceived behavioral control (*β* = 0.264) significantly predicted entrepreneurial intention. In contrast, the path from subjective norm to intention was not statistically significant. However, subjective norm had significant indirect effects, as it positively influenced both personal attitude and perceived control. Based on these findings, Liñán and Chen recommended that future research examine the indirect role of subjective norms in shaping entrepreneurial intention. With respect to demographic controls, the study found that gender, work experience, and the presence of a role model had small but significant effects on the TPB constructs, whereas age and nationality did not show significant associations.

Other similar works in the field of entrepreneurial intent have also found strong influences of personal attitude and perceived behavioral control on entrepreneurial intent but weak or not significant effects of demographic variables or among subjective norm and entrepreneurial intent (Autio et al., 2001; Krueger et al., 2000; Izquierdo & Buelens, 2011). However, other studies have found a relevant impact of subjective norm on entrepreneurial intent (Kolvereid & Isaksen, 2006), as it may seem that supportive environments increase entrepreneurial intent through self-efficacy, where people thrive in contexts with more resources and opportunities, as well as with fewer perceived obstacles to reach entrepreneurial success (Chen et al., 1998; Krueger et al., 2000; Drnovsek & Erikson, 2005).

### 1.2. Academic Entrepreneurial Intention (AEI)

Also drawing on the Theory of Planned Behavior (Ajzen, 1991), several studies across diverse contexts have examined how TPB constructs influence Academic Entrepreneurial Intention (AEI), which in this study is treated as a proxy for translational mindset.

A number of studies have tested the TPB model with academic populations, consistently highlighting the central role of personal attitude and perceived behavioral control. For example, in a study of German scientists, Goethner et al. (2012) found that personal attitude (*β* = 0.31) and perceived behavioral control (*β* = 0.14) significantly predicted AEI, while social norm was not a significant direct predictor. Indirectly, however, social norm influenced AEI through its effects on the other two TPB constructs. Their model also included economic predictors, such as expected reputational and financial benefits, which had indirect effects on intention, as well as human and social capital factors like cooperation with industry (*β* = 0.26) and public support institutions (*β* = 0.25), which shaped perceived behavioral control.

Similarly, Terán-Pérez et al. (2021) investigated both TPB and individual-level antecedents (e.g., creativity, entrepreneurship training) in a sample of Mexican university academics. Their SEM model showed strong direct effects from personal attitude (*β* = 0.607) and perceived behavioral control (*β* = 0.248), while social norm again was not a significant direct predictor. Among individual factors, perceived utility (β = 0.504) and creativity (*β* = 0.298) had the strongest effects on TPB components. For this reason, the authors highlight the importance of institutional infrastructure—such as training, science parks, and technology transfer offices—in fostering researchers’ attitudes and confidence toward entrepreneurial activity.

Other studies confirm the relative strength of personal attitude and perceived control. In a sample of Malaysian academics, Anuar et al. (2018) found all three TPB constructs to be significantly correlated with commercialization intention, with personal attitude showing the strongest relationship (*r* = 0.598, *p* < .001). Similarly, Arzenšek et al. (2018), using multiple regression with Slovenian doctoral candidates, reported personal attitude (*β* = 0.787) and subjective norm (*β* = 0.324) as significant predictors of intention to collaborate on applied research projects. In this case, perceived behavioral control was not a significant predictor.

Environmental and institutional factors have also been found to moderate or reinforce TPB relationships. Feola et al. (2017), using SEM with STEM European doctoral students, found that perceived behavioral control (*β* = 0.495) had the strongest direct effect on AEI, followed by subjective norm (*β* = 0.240) and personal attitude (*β* = 0.162). University support (*β* = 0.361) and government support (*β* = 0.323) were the strongest predictors of personal attitude, while financial and industrial support directly influenced AEI (*β* = 0.434).

In a related study, Samo and Huda (2019) examined institutional, governmental, and industry support among early-career researchers in Pakistan. They found that all three factors influenced AEI, with the strongest direct effect emerging from academic settings (*β* = 0.421). As in Terán-Pérez et al. (2021), these findings underscore the role of joint efforts between universities and governments—such as incubators, research centers, and entrepreneurship-focused curricula—in supporting researchers’ engagement with AEI.

Contextual influences—ranging from institutional structures to sociocultural norms—have been shown to significantly shape academic entrepreneurial intention. Perkmann et al. (2013), in their systematic review of academic engagement, found that researchers’ involvement in entrepreneurial activities was associated with a range of demographic and organizational factors. Male and scientifically productive researchers were more likely to engage in both academic engagement and entrepreneurship, with younger researchers more involved in commercialization and senior academics more successful in securing grants. Engagement was also higher in institutions with strong research profiles, formal technology transfer mechanisms, and entrepreneurial departmental cultures. Disciplinary context played a role as well, with researchers in fields such as biomedical and chemical engineering more likely to engage in entrepreneurship. Complementing these findings, Tarapuez-Chamorro et al. (2020) reported that subjective norm significantly influenced AEI in Colombia, driven by admiration for entrepreneurs and a desire for autonomy. Similarly, Kickul and Zaper (2000) found that proactive individuals were more likely to pursue entrepreneurial paths when not constrained by institutional obligations. In academia, such obligations may include tenure-track commitments that reduce perceived flexibility, potentially discouraging translational engagement. Taken together, these findings highlight the complex interplay between organizational, disciplinary, and cultural contexts in shaping how researchers engage with entrepreneurial and translational behaviors.

Together, these studies demonstrate the strong predictive value of personal attitude and perceived behavioral control across entrepreneurial contexts, while also highlighting the role of institutional, disciplinary, and cultural environments. Yet, despite their contributions, existing models remain limited in scope and language, reinforcing the need for frameworks—such as translational mindset—that better reflect how academics conceptualize applied impact beyond commercialization.

## 2. Methods

### 2.1. Participants

This study analyzed data from 257 researchers and graduate students affiliated with R1 and R2 institutions across the United States. Participants were recruited based on their involvement in engineering, medical, or biological sciences, ensuring a sample reflective of individuals working at the intersection of academic research and industry applications.

The sample included 65% men and 35% women, spanning diverse career stages. Regarding age, 81% were under 45, including 2% aged 18–24, 30% aged 25–34, and 49% aged 35–44. The remaining 19% were 45 or older, with 13% aged 45–54, 4% aged 55–64, and 2% aged 65 and above.

Academic backgrounds varied, with 30% holding a doctoral degree, 64% a master’s degree, and 6% a bachelor’s degree. In terms of field of specialization, 43% worked in engineering, 34% in medical or biological sciences, and 23% in physical sciences. Institutional affiliation was balanced, with 44% from R1 (Very High Research Activity) institutions, 54% from R2 (High Research Activity) institutions, and 2% in other type of academic institutions. Participants held various roles within research environments, including faculty (38%), principal investigators (8%), graduate students (31%), and recent graduates (within the last five years; 22%).

Racial and ethnic representation was diverse, with 61% identifying as White, 14% as Black or African American, 12% as Asian, and 7% as Hispanic or Latino. Other ethnic groups, including Native Hawaiian or Pacific Islander, Middle Eastern or North African, and Multi-ethnic individuals, made up 6% of the sample.

Participants were recruited through InnovateMR, a research panel specializing in reaching academic and industry professionals. InnovateMR utilizes strict quality control measures (e.g., engagement monitoring, response tiem analysis, and attention checks) to ensure broad representation across demographics while maintaining data quality (InnovateMR, 2017; InnovateMR, 2024). As confirmed by the present data set, utilizing research panels for crowdsourcing provides diverse and reliable data (Behrend et al., 2011; Sumner et al., 2020) while still allowing for engagement with nuanced populations.

Outliers were identified using Mahalanobis and Cook’s distance criteria. However, as these cases represent genuine variability rather than data errors, they were retained to preserve the full range of responses in the sample.

### 2.2. Measure

The Translational Mindset Scale (TMS) (Elizondo, Murray, & Emler, 2025) was used to measure attitudes and beliefs about translating academic research into industry applications, as an adaptation to academic settings from the Entrepreneurship Intention Questionnaire (EIQ) (Liñán & Chen, 2009). An earlier version (Liñán & Chen, 2006) used the terms Personal Attraction, Perceived Social Norms, and Self-Efficacy—language retained in the present study to reflect the translation-specific context, though the constructs align with personal attitude, subjective norm, and perceived behavioral control in the final version of their model.

The scale included 22 items subdivided into four latent constructs, each assessed using a 7-point Likert scale:

- Personal Attraction: Interest in and motivation for research engagement and translation.
- Self-Efficacy: Confidence in one’s ability to translate research into industry applications.
- Perceived Social Norms: Perceptions of external expectations and approval for engaging in translation efforts.
- Translation Intention: The likelihood of actively engaging in knowledge translation.

Higher scores indicated stronger endorsement of translation-related attitudes and intentions.

The survey was administered online, and informed consent was obtained before participation. Participants were compensated for their time in accordance with InnovateMR’s compensation system. The study was conducted under the University of Kansas IRB approval (STUDY00151526), ensuring compliance with ethical research standards.

### 2.3. Analytical strategy

Structural Equation Modeling (SEM) was used to examine the hypothesized relationships among Personal Attraction (PA), Self-Efficacy (SE), Perceived Social Norms (PSN), and Translation Intention (TI) while accounting for potential confounding effects of demographic and institutional factors, based on the SEM model suggested by Liñén and Chen (2009). The model was estimated using lavaan (R software). The model tested a series of 28 hypotheses:

Direct Effects:

- H1: PA positively influences TI.
- H2: SE positively influences TI.
- H3: PSN positively influences TI.
- H4: PSN positively influences PA.
- H5: PSN positively influences SE.

Indirect Effects:

- H6: PSN indirectly influences TI through PA.
- H7: PSN indirectly influences TI through SE.

Control Variable Effects:

- H8, H9, H10: Gender directly affects PA, PSN, and SE.
- H11, H12, H13: Role at the university directly affects PA, PSN, and SE.
- H14, H15, H16: University type directly affects PA, PSN, and SE.
- H17, H18, H19: Age directly affects PA, PSN, and SE.
- H20, H21, H22: Education level directly affects PA, PSN, and SE.
- H23, H24, H25: Ethnicity directly affects PA, PSN, and SE.
- H26, H27, H28: Research area directly affects PA, PSN, and SE.

**Figure 1:**
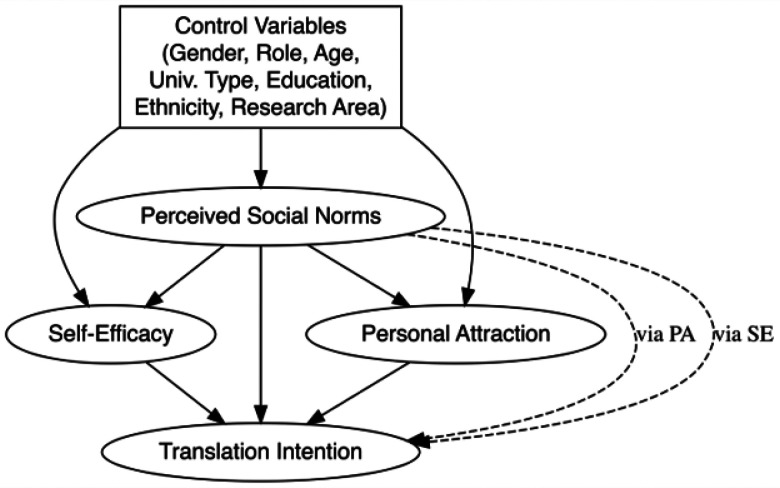
Hypothesized TMS model

The hypothesized structural model (Figure 1) included latent variables for PA, SE, PSN, and TI, each measured by multiple observed indicators (3–6 items), which group as four-correlated factors using Confirmatory Factor Analysis. In a previous study, the standardized factor correlations ranged from 0.677 to 0.897, indicating strong conceptual relationships among constructs. Specifically, Self-Efficacy and Translation Intention were most strongly correlated (β=0.897), while Perceived Social Norms showed moderate correlations with both Self-Efficacy (β=0.677) and Personal Attraction (β=0.814) (Elizondo, Murray, & Emler, 2025).

The effects of demographic and institutional factors (gender, role at university, institution type, age, education, ethnicity, and research area) were included as exogenous control variables[1], with direct paths specified to PA, PSN, and SE. Model fit was assessed using multiple fit indices, including the Comparative Fit Index (CFI), Tucker-Lewis Index (TLI), Root Mean Square Error of Approximation (RMSEA), and Standardized Root Mean Square Residual (SRMR) where the model is expected to meet commonly recommended thresholds (CFI and TLI > 0.90, RMSEA and SRMR < 0.08; Hu & Bentler, 1999). To account for potential non-normality in the data, robust standard errors were used for the estimation of direct effects and model fit indices. Bootstrapped standard errors and p-values based on 1,000 resamples were used for indirect effect testing. All analyses were conducted in R (version 4.4.2).

[1] In all models estimated using lavaan, categorical control variables were dummy-coded as follows: Field of Study (Medical/Biological Sciences = 0, Other = 1), Education Level (Doctoral = 0, Other = 1), Institution Type (R1 = 0, R2 = 1), Gender (Male = 0, Other = 1), Age (≤44 years = 0, 45+ = 1), Role (Faculty=0, Other = 1), and Ethnicity (White = 0, Non-White = 1).

## 3. Results

The hypothesized structural equation model (SEM) demonstrated an adequate fit (Table 1). Bootstrapped standard errors and confidence intervals were used to ensure robust parameter estimation. These results suggest that the specified relationships among latent constructs provide a reasonable fit to the data.

The structural equation model tested multiple hypotheses regarding the relationships among Personal Attraction, Self-efficacy, Perceived Social Norms, and external factors. As shown in Table 2, most hypothesized relationships were supported. However, some predictors, such as gender, university type, and field, did not show significant effects on key constructs.

### 3.1. Predictors of Translation Intention

Personal Attraction was the strongest predictor of Translation Intention, indicating translation intention is highly associated with individual personal traits and motivations. Self-Efficacy also showed a strong positive effect, suggesting that confidence in one’s ability to conduct and apply research is closely linked to engagement in knowledge translation. Perceived Social Norms, in contrast, had a statistically significant negative direct effect on Translation Intention, implying that stronger external expectations may actually discourage translational behavior. However, this does not negate the role of social norms entirely. While the indirect effect of Perceived Social Norms through Personal Attraction was not statistically significant, a significant indirect path through Self-Efficacy did emerge. This suggests that external expectations can still promote research translation, but primarily when they enhance individuals’ self-confidence in their research capabilities.

### 3.2. Predictors of Personal Attraction, Self-Efficacy, and Perceived Social Norms

Perceived Social Norms significantly predicted both Personal Attraction and Self-Efficacy, indicating that individuals who perceive stronger research-related expectations are more motivated and confident in conducting and applying research. Self-Efficacy was also positively associated with being at an R1 institution, whereas participants at R2 institutions reported lower confidence. Additionally, non-faculty participants perceived significantly stronger social norms around research engagement than faculty.

### 3.3. Non-Significant Predictors (No Effect Found)

No significant differences in Personal Attraction, Self-Efficacy, or Perceived Social Norms were found based on gender, age, education level, or ethnicity. However, descriptive trends indicate that women, participants from R2 universities, and individuals over 45 years of age tended to report lower translation intention, whereas participants in engineering and physical sciences reported higher translation intention compared to those in medical or biological sciences.

### 3.4. Model Re-specification

To enhance parsimony and improve interpretability, the model was respecified by removing non-significant paths. Global fit indices were reassessed to evaluate overall model performance, and the effect sizes of the remaining paths are reported. The updated model diagram reflects the final structure. The re-specified structural equation model demonstrated a similar, suitable fit as the hypothesized model (Table 3), still meeting the recommended global fit thresholds (Hu & Bentler, 1999).

Table 4 presents the means, standard deviations, and intercorrelations among the four latent constructs derived from the final SEM model. All variables showed moderately high average levels, with Translation Intention (TI) and Perceived Social Norms (PSN) having the highest means. Pearson correlations based on SEM-derived factor scores showed strong and statistically significant associations among all constructs (*p* < .001), with the strongest correlation observed between Self-Efficacy and Translation Intention (*r* = .93), and the weakest between Personal Attraction and Self-Efficacy (*r* = .76).

In the re-specified model, all observed indicators loaded significantly onto their respective latent constructs (*p* < 0.001), with standardized factor loadings ranging from 0.371 to 0.850. For the Personal Attraction factor, λ loadings ranged from 0.371 to 0.829, while Perceived Social Norms loadings ranged from 0.619 to 0.735. Self-Efficacy items demonstrated strong loadings, ranging from 0.755 to 0.843, and Translation Intention items loaded between 0.800 and 0.850. Overall, the pattern of loadings in Table 5 supports acceptable to strong convergent validity for the measurement components of the model.

As for the relationships among Personal Attraction, Self-efficacy, Perceived Social Norms, and external factors in the re-specified model, Table 6 shows that all the hypotheses were supported at the 0.05 alpha level.

**Figure 2:**
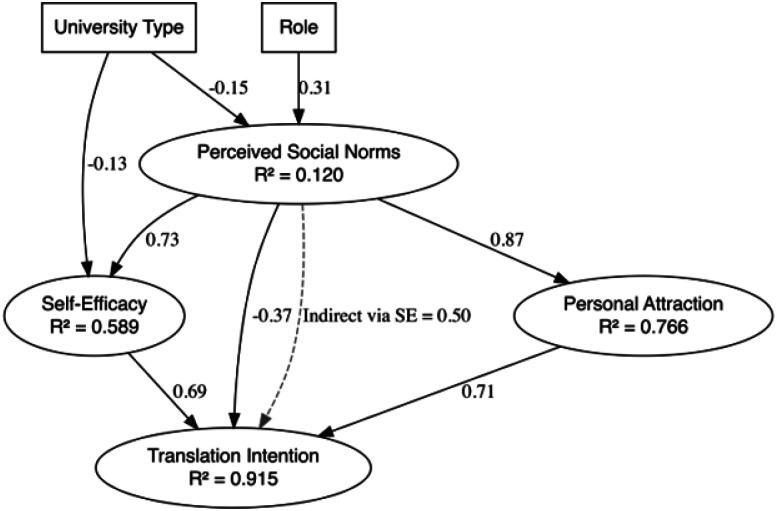
Final SEM TMS model *Note*. Standardized path coefficients are shown. Solid lines represent significant direct effects; the dashed lines indicate significant indirect effects.

The re-specified model (Fig. 2) explained a substantial proportion of variance in the key latent constructs. Specifically, the model accounted for 91.5% of the variance in Translation Intention, 76.6% in Personal Attraction, 58.9% in Self-Efficacy, and 12.0% in Perceived Social Norms. These values indicate strong explanatory power for the primary outcome and motivational constructs. Although Perceived Social Norms had a negative direct effect on Translation Intention, it also contributed indirectly through Self-Efficacy, highlighting its complex role in shaping translational engagement. Perceived Social Norms remained a strong predictor of both Self-Efficacy and Personal Attraction.

Two background variables were retained in the final model: university type affiliation and role. Participants from R2 institutions reported lower Self-Efficacy and perceived weaker Perceived Social Norms compared to those from R1 institutions. Additionally, non-faculty participants reported stronger Perceived Social Norms than faculty.

It should be noted that measurement invariance was assessed using multi-group CFA across all demographic groups. Scalar invariance was supported for role, education, age, and ethnicity, permitting meaningful comparisons across these groups. For university type, gender, and field, only configural invariance was achieved. As such, while university type showed significant effects in the final SEM model, these should be interpreted with caution.

## 4. Discussion

This study examined the structural relationships underlying the Translational Mindset Scale (TMS), which was adapted from an early draft of Liñán and Chen’s (2009) entrepreneurial intention model. The goal was to test whether the constructs in the scale would operate similarly when applied to the intention to engage in research translation.

Aligning with previous findings (Liñán & Chen, 2009; Izquierdo & Buelens, 2011; Goethner et at., 2012; Anuar et al., 2018; Arzenšek et al., 2018; Terán-Pérez et al., 2021), Personal Attraction was the strongest predictor of Translation Intention. However, a key difference emerged in the role of Perceived Social Norms. While these authors found no significant direct effect of subjective norms on entrepreneurial intention and did not estimate indirect effects, they did suggest that future studies should explore possible mediating paths. This study addressed that gap by showing that social expectations worked in two ways: on their own, they lowered people’s intention to engage in research translation, but when they helped people feel more confident, they had the opposite effect. Translation was more likely when social pressure translated into personal belief, aligning with other studies (Chen et al., 1998; Krueger et al., 2000; Drnovsek & Erikson, 2005). In this way, the current findings extend the original model and offer a more nuanced account of how external influences operate within academic research contexts.

The contrast between the full theoretical model (Fig. 1) and the final respecified model (Fig. 2) offers a clearer picture of how the structural factors interact in shaping Translation Intention. While the original model incorporated a comprehensive set of background variables, only role (faculty, staff, etc.) and university type (R1 vs. R2) remained as meaningful predictors, influencing Perceived Social Norms and Self-Efficacy. Specifically, participants from R2 institutions perceived weaker social norms than those at R1 institutions, while non-faculty participants—including graduate students and principal investigators—reported stronger perceptions of translational expectations compared to faculty. This finding suggests that institutional context and professional identity may be more influential than personal demographics in shaping how researchers perceive expectations around translation, supporting previous findings (Kickul & Zaper, 2000; Terán-Pérez et al., 2021). Similarly, this adds weight to the argument that efforts to foster research translation should consider organizational climate and professional development, rather than relying solely on interventions targeted at individuals based on background traits (Feola et al., 2017).

## 5. Conclusion and limitations

This study contributes to the growing literature on research translation by validating the Translational Mindset Scale (TMS) and testing a theory-driven structural model adapted from early versions of Linán and Chen’s (2009) entrepreneurial intention framework. The findings confirm that Personal Attraction and Self-Efficacy are central to researchers’ intention to engage in translational activity, while Perceived Social Norms play a more complex role, indirectly supporting intention when they enhance confidence. Notably, the study also clarifies the importance of institutional context and professional identity over individual demographics in shaping key motivational constructs. This reinforces the idea that efforts to foster translation should move beyond individual-level training and instead invest in creating supportive environments, mentorship structures, and institutional incentives that strengthen researchers’ self-belief and sense of alignment with translational goals.

This study is limited by its reliance on self-reported data and its U.S.-based sample, which included STEM researchers from R1 and R2 institutions. While diverse in roles and disciplines, the sample may not represent other academic or international contexts. Most background variables were included as part of an exploratory model and did not remain in the final structure, underscoring the need for replication with broader and more varied populations. Finally, as the model focuses on intention rather than behavior, future research should examine how these motivational constructs translate into actual translational activity. Longitudinal and cross-cultural studies will be essential to refine the TMS and better understand how institutional and psychological factors interact across settings.

## Figures and Tables

**Table 1:** Global fit indices for hypothesized SEM model

*χ^2^*	*df*	*p*	CFI	TLI	RMSEA	SRMR
598.284	337	< 0.001	0.932	0.923	0.055	0.051

**Table 2:** Hypothesis testing results for hypothesized SEM

Hypothesis	*β*	SE	*p*	Decision
***Direct effects***				
H1: Personal Attraction → Translation Intention	0.696	0.470	<0.001	Supported
H2: Self-Efficacy → Translation Intention	0.686	0.114	<0.001	Supported
H3: Perceived Social Norms → Translation Intention	−0.355	0.208	0.030	Supported
H4: Perceived Social Norms → Personal Attraction	0.850	0.090	<0.001	Supported
H5: Perceived Social Norms → Self-Efficacy	0.700	0.104	<0.001	Supported
*Indirect effects*				
H6: PSN → Personal Attraction → Translation Int.	0.592	0.418	0.072	Not Supported
H7: PSN → Self-Efficacy → Translation Int.	0.480	0.143	<0.001	Supported
*Control variables*				
H8: Gender → Personal Attraction	−0.021	0.051	0.689	Not Supported
H9: Gender → Perceived Social Norms	−0.013	0.121	0.840	Not Supported
H10: Gender → Self-Efficacy	0.061	0.113	0.233	Not Supported
H11: Role → Personal Attraction	0.028	0.052	0.605	Not Supported
H12: Role → Perceived Social Norms	0.238	0.145	0.003	Supported
H13: Role → Self-Efficacy	0.036	0.120	0.520	Not Supported
H14: University Type → Personal Attraction	0.045	0.045	0.340	Not Supported
H15: University Type → Perceived Social Norms	−0.185	0.133	0.014	Supported
H16: University Type → Self-Efficacy	−0.139	0.113	0.009	Supported
H17: Age → Personal Attraction	−0.052	0.071	0.384	Not Supported
H18: Age → Perceived Social Norms	−0.040	0.200	0.650	Not Supported
H19: Age → Self-Efficacy	0.012	0.160	0.194	Not Supported
H20: Education → Personal Attraction	0.053	0.051	0.289	Not Supported
H21: Education → Perceived Social Norms	0.102	0.140	0.159	Not Supported
H22: Education → Self-Efficacy	0.071	0.120	0.172	Not Supported
H23: Ethnic → Personal Attraction	0.058	0.042	0.181	Not Supported
H24: Ethnic → Perceived Social Norms	0.069	0.132	0.346	Not Supported
H25: Ethnic → Self-Efficacy	0.063	0.103	0.191	Not Supported
H26: Field → Personal Attraction	0.008	0.044	0.849	Not Supported
H27: Field → Perceived Social Norms	0.036	0.126	0.600	Not Supported
H28: Field → Self-Efficacy	0.078	0.122	0.155	Not Supported

*Note*. *β* = Standardized coefficient, SE = Standard Error.

**Table 3:** Global fit indices for re-specified SEM model

*χ^2^*	*df*	*p*	CFI	TLI	RMSEA	SRMR
485.214	275	< 0.001	0.937	0.930	0.062	0.056

**Table 4:** Means, Standard Deviations, and Correlations Among TMS Constructs

Variable	*M*	*SD*	PA	SE	PSN	TI
Personal attraction (PA)	5.81	0.87	1	0.76***	0.93***	0.89***
Self-Efficacy (SE)	5.48	1.13		1	0.82***	0.93***
Perceived Social Norms (PSN)	5.99	0.88			1	0.83***
Translation Intention (TI)	5.71	1.14				1

*Note*. *M* = mean, *SD* = standard deviation. All correlations are based on factor scores extracted via regression method from the SEM model.

*** *p* < 0.001.

**Table 5:** Factor loadings in re-specified SEM model

Latent Variable	Item	Std. Loading
Personal Attraction	Q1R1	0.371
	Q1R2	0.786
	Q1R3	0.525
	Q1R4	0.653
	Q2R1	0.835
	Q2R2	0.829
	Q2R3	0.703
Perceived Social Norms	Q3R1	0.735
	Q3R2	0.685
	Q3R3	0.682
	Q3R4	0.619
Self-Efficacy	Q4R1	0.755
	Q4R2	0.843
	Q4R3	0.818
	Q4R4	0.839
	Q4R5	0.788
	Q4R6	0.753
Translation Intention	Q5R1	0.826
	Q5R2	0.840
	Q5R3	0.850
	Q5R4	0.800
	Q5R5	0.850

*Note*. All factor loadings are standardized estimates and statistically significant at *p* < 0.001.

**Table 6:** Hypothesis testing results for re-specified SEM

Hypothesis	*β*	SE	*p*	Decision
***Direct effects***				
H1: Personal Attraction → Translation Intention	0.711	0.479	<0.001	Supported
H2: Self-Efficacy → Translation Intention	0.687	0.112	<0.001	Supported
H3: Perceived Social Norms → Translation Intention	−0.371	0.220	0.031	Supported
H4: Perceived Social Norms → Personal Attraction	0.875	0.092	<0.001	Supported
H5: Perceived Social Norms → Self-Efficacy	0.735	0.110	<0.001	Supported
*Indirect effects*				
H7: PSN →Self-Efficacy →Translation Int.	0.505	0.135	<0.001	Supported
*Control variables*				
H12: Role → Perceived Social Norms	0.309	0.123	<0.001	Supported
H15: University Type → Perceived Social Norms	−0.153	0.120	0.025	Supported
H16: University Type → Self-Efficacy	−0.134	0.102	0.006	Supported
